# Inflight leg cuff test does not identify the risk for orthostatic hypotension after long-duration spaceflight

**DOI:** 10.1038/s41526-019-0082-3

**Published:** 2019-10-11

**Authors:** Katelyn N. Wood, Kevin R. Murray, Danielle K. Greaves, Richard L. Hughson

**Affiliations:** Schlegel-University of Waterloo Research Institute for Aging, Waterloo, ON Canada

**Keywords:** Physiology, Predictive markers

## Abstract

Landing day symptoms from orthostatic hypotension after prolonged spaceflight can be debilitating, but severity of these symptoms can be unpredictable and highly individual. We tested the hypothesis that an impaired baroreflex response to an inflight leg cuff test could predict orthostatic intolerance on return to Earth. Eight male astronauts (44 ± 7 years of age (mean ± SD); mean mission length: 167 ± 12 days) participated in a standardized supine-to-sit-to-stand test (5 min–30s–3 min) pre- and postflight, and a 3 min thigh cuff occlusion test pre- and inflight with continuous monitoring of heart rate and arterial blood pressure. The arterial baroreflex was not changed inflight as shown by similar reductions in mean arterial pressure (MAP) response to leg cuff deflation (preflight −19 ± 2 mmHg vs. inflight −18 ± 5 mmHg). With the sit/stand test, the nadir of MAP was lower postflight (−17 ± 9 mmHg) than preflight (−11 ± 6 mmHg, *p* < 0.05). A greater increase in heart rate (25 ± 7; 16 ± 3 bpm) and decrease in stroke volume (−24 ± 11; −6 ± 4 mL) occurred with sit/stand postflight than leg cuffs inflight (*p* < 0.001). Inflight testing was influenced by elevated cardiac output resulting in a smaller drop in total peripheral resistance. Two of eight subjects exhibited orthostatic hypotension during the postflight stand test; their responses were not predicted by the inflight leg cuff deflation test. These results suggest that the baroreflex response examined by inflight leg cuff deflation was not a reliable indicator of postflight stand responses.

## Introduction

Orthostatic intolerance, a major problem for the return of astronauts to the gravitational forces of Earth,^1,2^ is aggravated by long-duration spaceflight.^3^ NASA included this important health risk in the Human Research Roadmap of problems that need to be resolved prior to future exploration missions (NASA Human Research Roadmap).^4^ Multiple mechanisms contribute to orthostatic intolerance on return to Earth including impaired venous properties that reduce return of blood to the heart,^5,6^ lower blood volume,^7^ diminished cardiac mass,^8,9^ reduced cardiac output,^10^ inadequate sympathetic nervous system-mediated vasoconstriction,^1,11–13^ and impairment of the arterial baroreflex.^14,15^

Based on the current knowledge and understanding of cardiovascular mechanisms that are altered during exposure to microgravity, a test, administered during spaceflight, to identify individual astronauts at greatest risk for postflight orthostatic hypotension could provide input guiding the extent of near end-of-flight and immediate postflight countermeasures designed to reduce the risk of impairment of performance and loss of consciousness. These end-of-flight countermeasures could include lower body negative pressure, as used by Russian cosmonauts, high intensity exercise, and fluid loading during re-entry; whole body cooling, and lower body compression garments alone or in combination, can provide protection against postflight orthostatic intolerance.^16^ Methods to challenge the arterial blood pressure (BP) regulatory mechanisms can be applied while in microgravity. A human centrifuge could generate a head-to-foot gravitational vector, but this facility is not currently available in space. Lower body negative pressure could stimulate the effects of gravity and retain blood volume in the lower part of the body. In the Russian segment of the International Space Station (ISS) the Chibis device generates lower body negative pressure, but this device was unavailable to the researchers at the time of this experiment. Leg cuff inflation to occlude arterial inflow followed by rapid release of the cuffs causes a drop in arterial BP that is similar to that of transition from supine to upright posture.^17,18^ This leg cuff test detected differences in arterial baroreflex response between endurance athletes and healthy controls, with the athletes experiencing a greater drop in BP and a longer time for recovery.^18^ It was hypothesized that astronauts who experienced postflight orthostatic intolerance during a stand test would have impairment of arterial baroreflex manifested by a greater drop and delayed recovery of arterial BP in the leg cuff test when measured late in spaceflight.

## Results

### Preflight posture

**Fig. 1:**
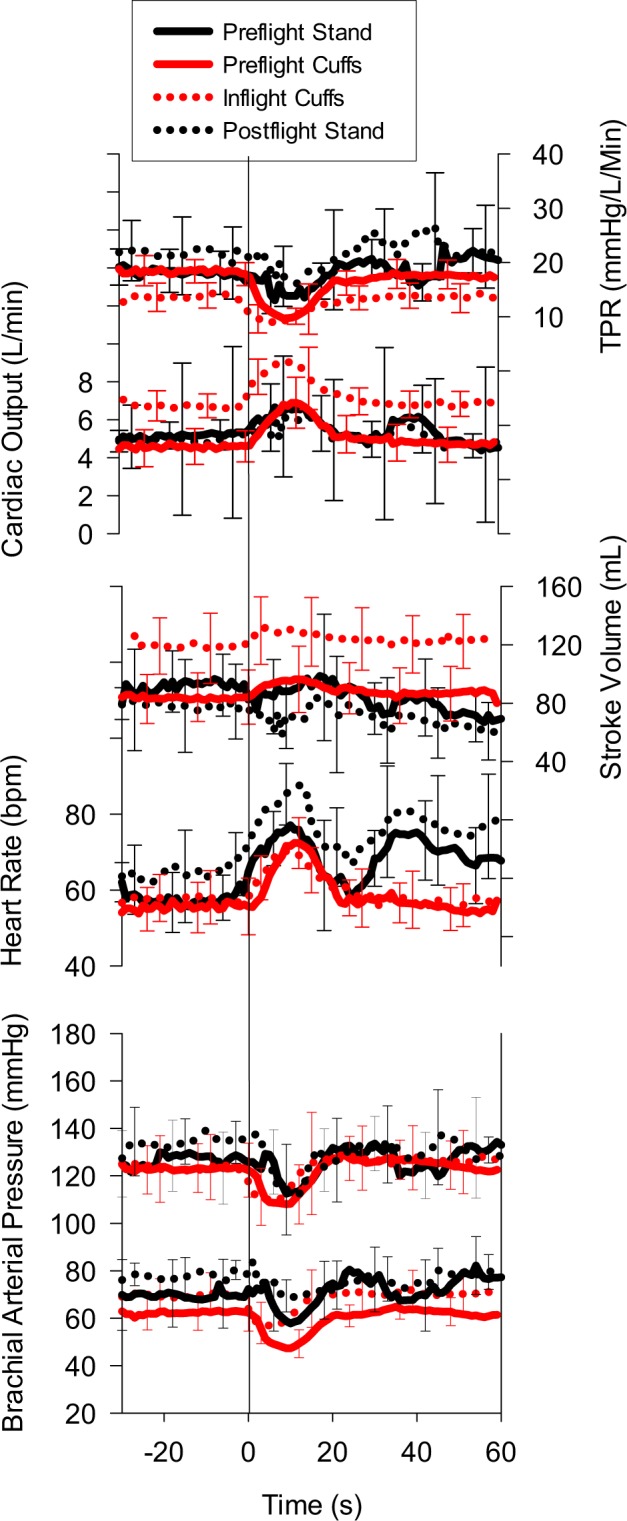
Cardiovascular response to posture (black) vs. leg cuffs (red) in all subjects, preflight (solid lines) and postflight/inflight (dotted lines). Solid vertical line denotes initiation of cuff deflation or moving from supine to seated. Values are means ± SD

The cardiovascular responses to the supine-to-seated-to-standing posture transitions displayed an anticipated biphasic response (solid black line, Fig. 1). Heart rate (HR) increased from supine to seated (*p* < 0.001) and remained elevated above supine values at the end of the 3 min stand (*p* < 0.01, Table 1). Systolic (SBP), diastolic (DBP), and mean arterial pressure (MAP) decreased from supine to seated (*p* < 0.05) with a gradual return to above supine values at end stand (Fig. 1, Table 1). Cardiac output (Q) increased from supine to seated (*p* < 0.001) and was slightly elevated at end stand (*p* = 0.06). Stroke volume (SV) decreased in the seated posture (*p* < 0.001) and remained lower at end stand (*p* < 0.05). Total peripheral resistance (TPR) decreased from supine to seated (*p* < 0.001) and was above supine values at end stand (*p* < 0.05).

**Table 1:** Cardiovascular variables for supine (SUP) to seated (SIT), to standing (STAND) posture pre- and postflight

	Pre-SUP	Pre-SIT	Pre-STAND	Pre-END STAND	Post-SUP	Post-SIT	Post-STAND	Post-END STAND
BRS, ms/mmHg	20.0 ± 5.6	NA	6.3 ± 3.0*	NA	19.1 ± 7.1	NA	7.4 ± 4.1*	NA
HR, bpm	57 ± 6	81 ± 11*	81 ± 11*	74 ± 11*	65 ± 8^+^	90 ± 10*^+^	88 ± 14*	86 ± 18*
SBP, mmHg	127 ± 14	101 ± 15*	106 ± 26	129 ± 12	134 ± 8	97 ± 25*	111 ± 27^~^	125 ± 11
DBP, mmHg	69 ± 8	54 ± 11*	61 ± 16^~^	78 ± 4*	78 ± 6^+^	54 ± 13*	63 ± 18*^~^	76 ± 9
MAP, mmHg	92 ± 7	73 ± 13*	80 ± 19	97 ± 6	98 ± 5	67 ± 18*	82 ± 20*^~^	93 ± 11
Q, L/min	5.2 ± 0.9	7.9 ± 2.3*	6.9 ± 2.2^~^	4.8 ± 0.8	5.0 ± 1.5	7.4 ± 3.3*	6.6 ± 2.7^~^	4.7 ± 1.2
SV, mL/min	93 ± 24	63 ± 26*	51 ± 20^~^	65 ± 16*	78 ± 25^+^	53 ± 27*	46 ± 21*	55 ± 13*
TPR	18.0 ± 5.0	10.5 ± 3.0*	13.4 ± 3.8*	20.4 ± 4.4*	21.6 ± 7.4	14.0 ± 8.3*	15.7 ± 7.8*^~^	21.1 ± 6.8

Values are ±SD. SUP is the mean of the 30 s supine period before moving to a seated position, SIT is the peak/nadir during the 30 s seated posture, STAND is the peak/nadir of the first 60 s of the stand, and END STAND is the mean value for the final 15 s of the stand test. *BRS* baroreflex slope (mean values shown); HR, heart rate (peak values shown), *SBP* systolic blood pressure (nadir values shown), *DBP*, diastolic blood pressure (nadir), *MAP* mean arterial pressure (nadir), *Q* cardiac output (peak), *SV* stroke volume (nadir), *TPR* total peripheral resistance (nadir)

*Different from sessional SUP

^~^Different from sessional SIT

^+^Different from corresponding preflight value, *p* < 0.05

### Postflight posture and comparison

Dotted black lines in Fig. 1 show the postflight posture transition responses. Compared to preflight, postflight supine HR was higher than preflight leading to a higher HR during the posture test (Table 1), yet the magnitude of change was not different (*p* = 0.45, Δ in Table 3). Supine arterial BP tended to be higher postflight than preflight, with a larger decrease (Δ) postflight on moving to the seated position for SBP. There was a longer time to nadir for DBP and MAP (*p* < 0.05, Table 3). There were no differences in the Q response pre- to postflight. Supine SV was lower postflight (*p* < 0.05), but the magnitude of change was the same when compared to preflight (*p* = 0.15). There were no differences in the TPR response pre- to postflight, despite a trend for higher postflight resistance.

### Preflight leg cuffs

The cardiovascular responses to the sudden release of occluding leg cuffs revealed the anticipated drop in arterial pressure and compensatory recovery responses (solid red line, Fig. 1). HR increased with cuff release (*p* < 0.001), while SBP, DBP, and MAP were reduced (*p* < 0.001, Table 2). On average, there was no significant change in SV with cuff release. Q increased with cuff release (*p* < 0.001), while TPR decreased (*p* < 0.001, Fig. 1, Table 2).

**Table 2:** Cardiovascular variables for leg cuff inflation-deflation pre- and inflight

	Pre-CUFF	Pre-Max or Min	Inflight CUFF	Inflight Max or Min
HR, bpm	56 ± 6	74 ± 7*	57 ± 6	74 ± 5*
SBP, mmHg	123 ± 11	107 ± 13*	123 ± 14	107 ± 16*
DBP, mmHg	62 ± 7	47 ± 7*	69 ± 8^+^	55 ± 9*^+^
MAP, mmHg	82 ± 8	63 ± 9*^+^	89 ± 10	71 ± 11*^+^
Q, L/min	4.6 ± 0.7	7.0 ± 1.4*	6.7 ± 1.2^+^	9.5 ± 2.3*^+^
SV, mL/min	84 ± 16	81 ± 15	119 ± 32^+^	115 ± 32*^+^
TPR, mmHg/L/min	18.4 ± 2.3	9.6 ± 1.6*	13.7 ± 3.5^+^	8.3 ± 2.6*

Values are mean ± SD. “Cuff” is near end of occlusion before the release of the thigh cuffs, ‘Max or Min’ is the maximal or minimal value after deflation. *HR* heart rate, *SBP* systolic blood pressure, *DBP* diastolic blood pressure, *MAP* mean arterial pressure, *Q* cardiac output, *SV* stroke volume, *TPR* total peripheral resistance. Note that Q and SV have been corrected for rebreathing estimate

*Different from sessional baseline during cuff inflation

^+^Different from corresponding pre-flight value, *p* < 0.05

### Inflight leg cuffs and comparison

The inflight leg cuff responses are shown by dotted red lines in Fig. 1. Spaceflight did not change HR, SBP, or MAP compared to preflight. DBP, SV, and Q were greater inflight (*p* < 0.05, Table 2). TPR was lower inflight (*p* < 0.05, Table 2). In addition, the magnitude of the response (Table 3) to cuff release was not different from preflight for HR, SBP, DBP, MAP, and Q. SV was higher inflight than preflight across all time points (Table 2), yet the magnitude of the reduction was the same compared to preflight (Table 3). TPR was lower inflight, and after the release of leg ischemia, the drop in TPR was smaller inflight (*p* < 0.01, Table 3) than preflight. There were no differences for any measured variable in the response rate to leg cuff deflation or the recovery rate (Table 3).

**Table 3:** Peak changes and response dynamics comparing preflight to postflight posture (SUPINE-SIT) and preflight to inflight leg cuff deflation (CUFF), respectively

	Pre-SUPINE-SIT	Post-SUPINE-SIT	Pre-CUFF	Inflight CUFF
Heart rate				
Change to peak (Δ bpm)	23.7 ± 7.6	25.2 ± 7.0	18.6 ± 7.2	16.4 ± 3.3
Time to peak (s)	9.1 ± 4.8	12.5 ± 1.1	10.3 ± 1.8	9.3 ± 3.4
Response rate (bpm/s)	4.4 ± 5.6	2.0 ± 0.5	1.9 ± 1.0	2.0 ± 0.9
Recovery rate (bpm/s)	−1.7 ± 1.3	−2.2 ± 1.0	−2.6 ± 1.2	−2.5 ± 2.8
Systolic blood pressure				
Change to peak (Δ mmHg)	−25.8 ± 19.0	−37.1 ± 25.9	−16.4 ± 3.7	−16.4 ± 5.6
Time to Nadir (s)	11.0 ± 7.2	15.8 ± 7.5*	7.8 ± 1.3	5.4 ± 3.5
Response rate (mmHg/s)	−3.5 ± 2.9	−2.9 ± 2.1	−2.2 ± 0.7	−4.7 ± 4.1
Recovery rate (mmHg/s)	−1.0 ± 0.4	−2.7 ± 2.5	−3.7 ± 1.3	−3.2 ± 1.4
Diastolic blood pressure				
Change to peak (Δ mmHg)	−15.2 ± 11.4	−23.8 ± 11.9*	−15.8 ± 3.4	−14.4 ± 3.2
Time to Nadir (s)	11.6 ± 7.3	13.9 ± 8.3	8.6 ± 1.4	5.9 ± 2.7
Response rate (mmHg/s)	−2.0 ± 2.1	−2.8 ± 3.0	−1.9 ± 0.5	−2.9 ± 1.3
Recovery rate (mmHg/s)	−2.6 ± 4.8	−1.8 ± 0.9	−1.8 ± 2.3	−3.2 ± 1.2
Mean blood pressure				
Change to peak (Δ mmHg)	−19.4 ± 16.5	−31.1 ± 17.9*	−18.9 ± 2.3	−17.9 ± 4.5
Time to Nadir (s)	9.9 ± 5.1	13.8 ± 8.8	7.5 ± 1.2	5.5 ± 3.5
Response rate (mmHg/s)	−2.4 ± 2.4	−3.6 ± 3.4	−2.6 ± 0.5	−5.5 ± 5.9
Recovery rate (mmHg/s)	−1.7 ± 2.0	−2.4 ± 1.4*	−3.1 ± 0.8	−3.9 ± 1.7
Cardiac output				
Change to peak (Δ L/min)	2.7 ± 1.8	2.4 ± 1.9	2.4 ± 1.1	2.8 ± 1.2
Time to peak (s)	8.9 ± 4.1	8.6 ± 4.9	10.1 ± 1.5	9.1 ± 4.0
Response rate (L/min/s)	0.3 ± 0.2	0.3 ± 0.2	0.3 ± 0.1	0.4 ± 0.2
Recovery rate (L/min/s)	−0.2 ± 0.2	−0.1 ± 0.1	−0.4 ± 0.2	−0.5 ± 0.2
Stroke volume				
Change to peak (Δ mL)	−30.2 ± 6.2	−24.3 ± 11.0	−9.4 ± 17.7	−5.6 ± 4.2
Time to Nadir (s)	21.3 ± 9.8	11.6 ± 10.4	9.0 ± 4.7	9.4 ± 8.7
Response rate (mL/min/s)	−2.3 ± 2.7	−4.5 ± 3.7	−1.3 ± 1.6	−1.7 ± 2.0
Recovery rate (mL/min/s)	−4.2 ± 3.9	−2.1 ± 1.9	−1.5 ± 2.2	−0.4 ± 1.2
Total peripheral resistance				
Change to peak (Δ mmHg/L/min)	−7.5 ± 6.6	−7.6 ± 2.6	−8.7 ± 2.0	−5.4 ± 1.1*
Time to Nadir (s)	12.6 ± 6.7	16.8 ± 10.6	8.6 ± 0.9	6.5 ± 3.9
Response rate (mmHg/L/min/s)	−0.8 ± 1.0	−0.8 ± 0.8	−1.0 ± 0.3	−1.2 ± 1.1
Recovery rate (mmHg/L/min/s)	−0.9 ± 1.1	−0.8 ± 0.2	−1.1 ± 0.3	−0.8 ± 0.2

Values are means ± SD. SUPINE-SIT is the peak/nadir during the 30 s sit period of the posture trial, CUFF is the maximal or minimal value after leg cuff deflation. Change to peak = Δ from baseline; negative values indicate a decrease. Response rate = Δ from baseline/time to nadir; recovery rate = 50% Δ/time for recovery

*Different from preflight, *p* < 0.05

### Arterial baroreflex and Blood pressure

**Fig. 2:**
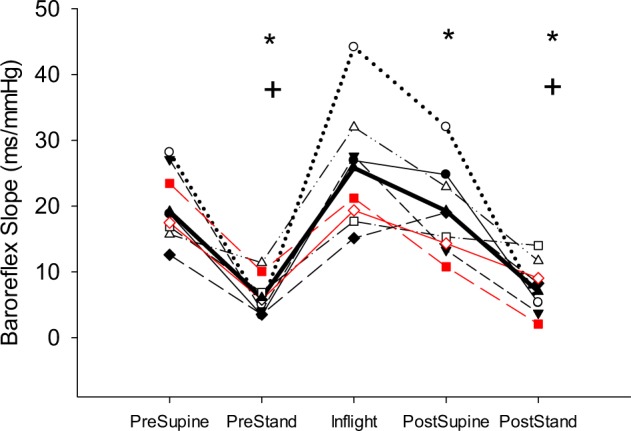
Individual subject values of the spontaneous arterial baroreflex slope from supine (pre- and postflight), standing (pre- and postflight), and inflight. Two subjects who exhibited signs of postflight orthostatic intolerance have been highlighted (red). Dark black line represents the group average (*n* = 8). *Different from inflight, *p* < 0.001; ^+^different from sessional supine, *p* < 0.001

The arterial baroreflex response slope was assessed inflight as well as pre- and postflight. The inflight baroreflex slope was significantly greater than preflight stand, and postflight supine and stand (*p* < 0.05, Fig. 2). Overall, there was a linear relationship between each individual’s baroreflex slope and his RR-interval, *r* ≥ 0.71 for all but one individual, and significant correlation coefficients for three individuals (*r* = > 0.96).

**Fig. 3:**
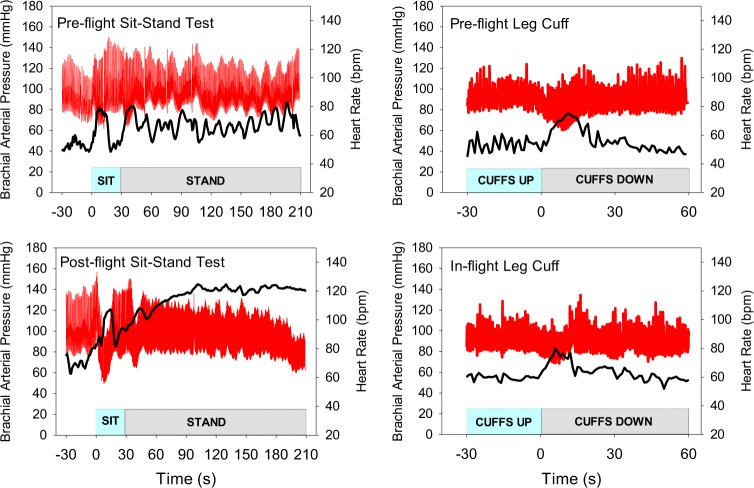
Continuous blood pressure (red) and heart rate (black) shown in one crew member who presented with orthostatic intolerance on return to Earth

While 6 of 8 subjects had remarkably stable BP during the postflight stand test, two subjects had patterns of BP consistent with orthostatic hypotension and suggestive of potential for syncope if the stand test had been sustained. Figure 3 demonstrates the pre- and postflight BP and HR response in one of the most vulnerable crewmembers. This figure also shows the leg cuff deflation tests revealing that the inflight leg cuff deflation test for this individual was not able to predict his postflight response to standing.

## Discussion

Two of eight male astronauts presented with orthostatic hypotension during a 3-min stand test conducted between 18 and 36 h of returning from 6 months on the ISS. Previous reports of orthostatic tolerance during a 10-min tilt test after short-duration shuttle flights found 20% of men and 100% of women to be intolerant.^19,20^ Meck et al.^3^ found that orthostatic testing by 10-min head-up tilt following long-duration spaceflight suggests a higher incidence rate (5 of 6 male astronauts) even though these astronauts had participated in inflight countermeasures while on the Mir space station. A compilation study of orthostatic tolerance after short-duration shuttle and long-duration stays on the ISS observed intolerance in 13 of 65 short-duration and 4 of 6 long-duration astronauts (sex of the intolerant astronauts was not specified).^20^ The primary hypothesis of this study, an inflight BP challenge caused by rapid deflation of leg cuffs, after a 3-min total arterial occlusion, would be predictive of those individuals most susceptible to postflight orthostatic hypotension, was not supported, but pointed to unique physiological adaptations to long-duration spaceflight that can influence predictive tests attempted in the future. Predictive tests, if they can be developed, could permit more aggressive near end of flight countermeasures for the 25% of male astronauts, and probably a greater proportion of female astronauts, at risk of impaired function due to cerebral hypoperfusion in the critical immediate postflight period.

Regulation of arterial BP in space might differ from on Earth. BP monitoring over 24-h revealed lower inflight values in short-^1,21^ and long-duration spaceflight.^22^ When measured under conditions similar to the current study, BP was not different from preflight.^23,24^ The mechanisms underlying the reported drop in arterial BP in 24-h recordings is unknown as the plasma catecholamine concentration was unchanged^22^ and direct recording of muscle sympathetic nerve activity was increased during the short-duration Neurolab mission.^25^ Lower BP in space might also reflect the overall reduction in physical activity.^26^ The resting supine DBP and MAP were slightly elevated inflight compared to preflight while the SBP was not different. With an increase in inflight SV, it was not expected that arterial pulse would be smaller, but the reduced TPR and greater flow through the splanchnic region^9^ might account for this. Consistent with the current study, the 45% increase in supine Q, corrected by the rebreathing value,^27^ was similar to values reported by Norsk et al.^22^

Leg cuff deflation provides a rapid drop in arterial BP enabling testing of arterial baroreflex^18^ and cerebrovascular function.^17^ This study is the first use of the leg cuff system during spaceflight to test BP regulation. With the elevated inflight Q, but unchanged BP, release of the leg occlusion cuffs caused reductions in SBP, DBP, and MAP that were not different between preflight and inflight testing. In addition to the similar drops in BP, the response and recovery rates from nadir of the BP were not different comparing preflight to inflight. Likewise, the HR response to the drop in arterial BP on cuff release was not different inflight as was the arterial baroreflex response slope not different inflight compared to preflight supine posture. Taken together, these data support the notion of maintenance of cardiovascular reflex control of heart rate and peripheral vascular resistance during long-duration spaceflight. These observations are consistent with measurements of arterial baroreflex during long-duration spaceflight,^23,24^ but contrast with reduced baroreflex responses during short-duration spaceflights.^15^

The similar drop in arterial BP on leg cuff release inflight compared to preflight indicated that the reduction in leg vascular resistance on cuff release was probably the same between the two tests. However, the change in TPR was less inflight compared to preflight because of the elevated inflight Q. Underlying the different TPR responses was a preferential distribution of blood flow to the splanchnic circulation reflected by the 45% increase in portal vein blood flow velocity,^28^ while blood flow velocity and vascular resistance in the muscular and cerebral circulations are not affected by spaceflight.^9^ We propose that the smaller drop in TPR resulted from the elevated Q where a similar reduction in leg vascular resistance on cuff release represented a smaller component of the whole-body TPR inflight. Further, with the elevated Q inflight, there was potential for a transient shift in blood flow from the splanchnic to the leg circulation without affecting the overall BP response.

Clinically relevant information on BP regulation is obtained from the supine-to-stand test^29^ and the sit-to-stand protocol.^30^ We included the 30-s sit phase of the study because we felt that the drop in arterial pressure on moving directly from supine-to-stand on return from spaceflight might expose astronauts to risk of fainting early after postural transition. The large drop in postflight BP in the seated posture for one astronaut (see Fig. 3), consistent with initial orthostatic hypotension,^29^ does support the response expected from typical clinical testing. For the group of astronauts, supine cardiovascular responses were different between pre- and postflight testing. HR was elevated, DBP was higher and SV was smaller in post- compared to preflight. The smaller SV might reflect a state of hypovolemia postflight, but the elevated HR maintained Q that combined with a slight elevation in TPR to keep MAP at, or slightly above, preflight supine. The elevation in DBP, with a consequent reduction in pulse pressure, reflected the smaller SV.

Active sitting from a supine position required muscular activity that compresses leg and splanchnic veins promoting increased venous return and Q.^31^ However, the muscular activity also caused arteriolar dilation resulting in reduced TPR, a fall in BP, and an increase in HR. Although not significant, there was a greater drop in SBP postflight than preflight during the supine to sitting transition. In addition, the time to nadir was longer postflight reflecting a delay in reaching this lower SBP. For DBP and MAP, the drop to nadir was also longer postflight. The response rate and the recovery rate determined as the time from supine to nadir and from nadir to 50% recovery of the drop in BP was not different except for the recovery rate of MAP. Individual variations in BP patterns limited the ability of these time course markers to reflect the integrated baroreflex response during the posture transition with this methodology.

Moving from seated to standing position activated the muscle pump assisting venous return and further dilating peripheral resistance vessels. With our protocol involving a 30-s period of seated posture between supine and standing, the magnitude of changes in cardiovascular variables appeared to be less than observed in sit-to-stand protocols.^30^ This was probably a consequence of incomplete recovery of the vasodilatory response to muscle contractions during the supine-to-seated transition. The absolute values of cardiovascular variables at the peak or nadir in the sitting-to-standing transition were not different between pre- and postflight testing. Likewise, there were no differences in the values measured in the last 15 s of the 3-min stand test from pre- to postflight, nor was there a difference in the baroreflex response slope during standing. These data suggest preservation of arterial BP regulation when measured within 18–36 h of return from 6 months in space. However, this conclusion from group data misses the two individuals with clear orthostatic hypotension during the 3-min stand. Figure 3 shows the astronaut with the greatest drop in BP. HR increased as anticipated suggesting that the cardiac component of the arterial baroreflex was intact, but at a HR of ~120 bpm, the sympathetic contribution would dominate the vagal component of the baroreflex as revealed by this individual’s response to postflight standing in Fig. 2. The progressive reduction in arterial pulse pressure (Fig. 3) is consistent with reduced venous return and smaller stroke volume; this type of response has been observed previously in some astronauts^32^ and is highly predictive of impending syncope if the test had been prolonged.

The BP responses of the astronaut in Fig. 3 can be used to assess the objective of the current study to utilize the leg-cuff deflation test to assess baroreflex response inflight as a predictor of postflight orthostatic hypotension. Inflight, the leg cuff deflation caused a similar, albeit slightly smaller, drop in BP and HR response. The rationale at the onset of the study was based on the observation that the preflight leg cuff test and the supine-to-seated transition caused similar drops in BP and increases in HR. However, the large drop in BP during the post-flight supine-to-seated transition was not reflected in the inflight leg cuff. The current data suggest that the elevated inflight Q changed the way in which TPR responded to the leg cuff deflation and therefore affected the integrated HR and vascular responses of the baroreflex. Inflight identification of up to 20–25% of all male astronauts^19,20^ and a greater percentage of female astronauts^19^ susceptible to postflight orthostatic hypotension is important for astronaut safety, but the approach needs to consider the elevated Q. Sustained movement of blood away from the heart, as in post-flight upright posture, is probably the best approach to assessing risk for orthostatic hypotension. Lower body negative pressure can progressively shift blood volume to the legs and lower abdomen evoking reflex cardiovascular responses. Currently, the Russian chibis would allow this; only limited data from Russian cosmonaut experiences with such tests have been published.^33^ A short-arm centrifuge could also accomplish a sustained gravity-like shift of fluid into the lower body, but this device is not available.

Astronaut research is unique because of the exposure to prolonged unloading conditions. Many factors complicate studies of physiological responses of astronauts to spaceflight and return to gravity. The most commonly referred to is the small sample size^34^ that also affected the current study. Different responses of male and female astronauts^19,35^ did not affect the current study, as all of our participants were men. Individual variability in preflight experiences and daily routine while on the ISS complicate interpretation of the varied physiological responses, especially in view of the small sample. Six of the eight astronauts in the current study were jet fighter pilots who would have experienced years of exposure to high gravitational stressors, and were selected for their occupation because they could tolerate high g-forces. It is possible that being a jet fighter pilot might have altered intrinsic physiological responses, or that unconscious responses contributed to their ability to maintain arterial BP. Unlike earlier short-duration missions on shuttle that frequently prioritized mission tasks over countermeasures, astronauts on ISS follow a monitored inflight countermeasures routine to attenuate cardiovascular deconditioning consisting of ~30 min/day aerobic exercise plus resistive exercises.^26,35,36^ The astronauts in the current study did not utilize the Russian chibis device to apply lower body negative pressure to achieve a gravity-like shift in blood volume. Most astronauts consume additional fluids and salt as part of the prereturn routine. Fluid loading might benefit orthostatic BP responses;^37^ but it is uncertain if this effect would have influenced our results obtained 18–36 h postflight when additional fluid consumption and hormonal responses would have contributed to plasma volume regulation.^6^ Further, physicians assess crewmembers immediately on return to Earth to determine if intravenous or oral fluid loading is required as astronaut health comes before science. Personal preferences and medical assessments influence whether the astronauts wear compression garments after reentry (Russian Kentavr). Only one astronaut in this study continued to wear Kentavr after landing, and removed them prior to testing.

In addition to the general limitations of spaceflight research considered above, the current study must acknowledge that estimates of stroke volume and Q from pulse contour analysis required careful positioning of the finger cuff and calibration. The pulse wave of the finger BP waveform was monitored in real-time by the principal investigator and assessed for quality against the preflight supine. Calibration of pulse contour Q was conducted preflight and inflight by comparison to rebreathing estimates; a major deviation in the calibration factor was detected inflight but the current data, obtained in a continuous data collection session with the rebreathing measurements, were adjusted by the appropriate factor.^27^ We did not do rebreathing postflight and used the preflight correction factors for each individual astronaut. This might have affected the absolute Q in the post-flight session. The pulse contour method has been used to monitor changes in Q.^38,39^ In the current study where all comparisons between conditions were with the same method, there might be an underestimation of the magnitude of change, but this should affect all conditions equally.

The stand test was limited to 3-min. The 3-min test is clinically relevant in diagnosis of classical orthostatic hypotension defined a drop in SBP > 20 mmHg or in DBP > 10 mmHg.^40^ Importantly in the context of spaceflight research, this duration test was appropriate due to the very limited time for multiple medical and research activities in the first day after spaceflight. All testing was conducted in what was considered “R + 0”, that is, the first day available for testing after landing. In some cases, astronauts arrived late at night at JSC and testing was completed as early as possible the next morning. The duration of the orthostatic challenge and time post-return are relevant.^3,20,41^ Previous investigations of post-spaceflight orthostatic intolerance used 10-min standing or tilt to identify orthostatic intolerance and testing was conducted in the context of shuttle return directly to the test site or after a shorter time to return to Star City, Russia.^3,20^ With the shorter duration of standing, the current study was unable to assess incidence of delayed orthostatic hypotension in astronauts after spaceflight.

In conclusion, postflight orthostatic hypotension remains a real risk for some astronauts returning to Earth after long-duration spaceflight. To date, it has not been possible to predict which astronauts will be most susceptible to the effects of orthostatic hypotension. The current study showed that the inflight response to rapid leg cuff deflation did not differ from the preflight response in terms of the magnitude or timing of the drop in arterial BP or the increase in HR. However, the inflight response did differ in the relative contribution of Q and TPR. That is, an elevated Q in the period immediately before cuff release allowed the drop in TPR as a consequence of the leg cuff release to result in a smaller change in TPR. Even in an astronaut with marked initial orthostatic hypotension with the post-flight transition to seated, the inflight immediate drop on cuff release did not predict this response. Future testing of astronauts to detect their potential for post-flight orthostatic intolerance needs to employ a sustained gravity-like challenge such as with lower body negative pressure.

## Methods

Eight male astronauts (44 ± 7 years of age) scheduled for a 6-month sojourn (mean mission length: 167 ± 12 days) to the ISS were recruited into the study following NASA and ESA informed consent procedures. The protocol was approved by the University of Waterloo Office of Research Ethics, the IRB, NASA Human Research Medical Review Board, the European Space Agency Medical Review Board and Japanese Space Agency Research Ethics board (NASA MPA 7116301606HR; FWA 00019876) in accordance with the Declaration of Helsinki. No alcohol, BP medications, over-the-counter cold medications, or allergy medications were consumed within 24 h of the test. Food and caffeine were not consumed in the 2 h before testing, and no exercise training was conducted on the test day before testing. Lower body compression garments, if worn, were removed for postflight testing.

Data collection consisted of four distinct phases of cardiovascular measurements: (1) preflight stand test, collected 44–90 days before launch for comparison to (2) postflight stand test, within 18–36 h of return to Earth when the astronauts were returned to Johnson Space Center, Houston TX, or the European Astronaut Centre, Cologne, Germany; (3) preflight supine leg cuff test, collected 44–90 days before launch for comparison to (4) inflight leg cuff, collected during the final 21 days of flight.

### Stand test

During preflight and postflight testing, crewmembers were instrumented in the supine posture to measure HR (Heart Rate Module; FMS, Amsterdam, The Netherlands) and finger arterial BP (Finometer Pro; FMS) with data recorded at 1000 Hz (PowerLab, ADInstruments). Finger pressure was referenced by a height correction system to the level of the right atrium on the midaxillary line. Following instrumentation requiring approximately 5 min, crewmembers rested in a supine position for collection of baseline data for 5 min before moving to a seated position with the legs bent at ~90° at the hip and knee. After being seated for 30 s, subjects were instructed to move as quickly as possible to a standing position while focusing on a target directly ahead and remaining stationary for 3 min before returning to a seated position. This test differed from a typical clinical test^40^ by introduction of a 30 s sit due to perceived higher risk of fainting with a supine to stand transition. The test adopted clinical criteria defining an acute drop in systolic BP greater than 40 mmHg or diastolic BP greater than 20 mmHg as initial orthostatic hypotension, and classical orthostatic hypotension as a drop in systolic BP greater than 20 mmHg, and/or diastolic BP greater than 10 mmHg over the 3 min.^29^

### On-ground leg cuffs

Crewmembers were instrumented with identical leg cuffs that were used on ISS (CADMOS Leg-Arm Cuff System, LACS). Following a 2-min stabilization period, leg cuffs were placed with the air bladder over the femoral artery and were manually inflated to suprasystolic pressure (150 mmHg) using two sphygmomanometers. The cuffs remained inflated for 3 min to occlude leg blood flow followed by the sudden release of the cuffs causing arterial pressure to transiently drop, as previously described by Lind-Holst et al.^18^ This protocol was repeated three times with a post-cuff period of 2 min between inflations. The responses to the three cuff tests were time aligned to the release of occlusion and averaged together before analysis.

### Rebreathing cardiac output

Following the leg cuff tests, crewmembers moved to a seated position for the rebreathing maneuvers to measure Q by a foreign gas technique using the Portable Pulmonary Function System (a ground-based equivalent of the Pulmonary Function System, PPFS; Danish Aerospace, Copenhagen, Denmark).^21^ Measurement of Q for these same astronauts was described previously.^27^ Participants were instructed to breathe normally through a mouthpiece, while wearing a nose clip. At the start of the rebreathing maneuver, they expired to a normal end-expiratory point and then followed a visual display that prompted smooth breathing back and forth to completely empty the bag at a rate of 20 breaths/min for 25 s. The bag contained 1.5 liter of a gas mixture [1% Freon-22 (soluble tracer gas), 1% SF_6_ (non-soluble tracer gas), 25% O_2_, and balance N_2_]. Three separate rebreathing maneuvers were separated by 5 min for elimination of the foreign gas markers.

### Inflight leg cuffs

This test was performed using the CADMOS LACS, a component of the ESA Cardiolab system located in the Columbus Module of the ISS. The LACS was computer controlled to inflate to the same suprasystolic pressure as on ground (150 mmHg) at the identical inflation rate as on ground, then to deflate rapidly to achieve a drop in BP. Arterial BP was measured using a continuous BP device (CBPD), which is part of the ESA Cardiolab. Astronauts instrumented themselves with finger cuffs from the CBPD, as well as leg cuffs on both upper thighs and performed three repetitions of the inflation/deflation cycle; as with preflight, the repetitions were time aligned and averaged prior to analysis. The analog CBPD data were transmitted to ground (via PPFS, Danish Aerospace) and visually monitored in real time by the PI in Waterloo, Canada. The PI was able to provide immediate feedback and have the crewmember adjust and correct finger cuff positioning as required. This procedure confirmed that the inflight leg cuff test produced BP waveforms that were qualitatively similar to those observed in preflight data collections.

All on-ground analog measures were sampled at 1000 Hz (inflight was 100 Hz, the maximum of the PFM) and stored for offline analysis (Lab Chart 7 software; AD Instruments). Pulse contour analysis of the finger arterial BP waveforms was completed in all cases by processing the digital waveform through the BeatScope program (FMS) that accepts only 200-Hz data sets, thus requiring a down-sampling of data from the pre-flight testing and an up-sampling by a sample and hold method of the 100-Hz data from the inflight testing to have a 200-Hz data set. Cardiac output using the PPFS was recorded at 100 Hz with the Agile Data Analyzer and Monitor (ADAM, Danish Aerospace).

We recently published data from these astronauts which demonstrated that Q estimated by pulse contour analysis was systematically different from rebreathing when comparing preflight to inflight data.^27^ Therefore, on-ground and inflight cardiac output values for each individual estimated by the Modelflow pulse contour analysis, were subsequently corrected by their own respective rebreathing values. As no rebreathing test was done in conjunction with the post-flight session, the postflight Modelflow data were corrected by the preflight factor.

Arterial baroreflex response slope was estimated from beat-to-beat RRI and SBP by applying the sequence method on approximately 240 consecutive cardiac cycles at rest and with postural change.^42,43^ Baroreflex sequences were defined by at least three consecutive beats in which the SBP and the RRI of the following beat either both increased or decreased. When a baroreflex sequence occurred, data pairs were analyzed with a linear regression. The slope of each individual sequence was computed, and the mean slope was determined as the average of all slopes within a given time period.

Data are expressed as means ± SD, unless otherwise noted. Statistical analysis was performed by repeated-measures analysis of variance using the Shapiro–Wilk test for normality and the Brown–Forsythe test for equal variance. The Holm–Sidak method was applied for multiple pairwise comparisons (SigmaPlot 13.0, Systat Software, Point Richmond, CA). For singular comparisons within conditions (i.e., supine to end stand), Student’s paired *t* test was applied when data were normally distributed; otherwise, the Mann–Whitney rank sum test was used. Differences were considered statistically significant when *P* < 0.05.

For the posture protocol, “supine” reflects the mean value for the 30 s period before moving to a seated position, “sit” reflects the peak/nadir of the 30-s seated period, “stand” reflects the peak/nadir of the first 60 s of the stand, and “end stand” reflects the mean value for the final 15 s of the stand test. For the leg cuff ischemia protocol, “cuff” reflects mean values 30 s before the release of the thigh cuffs, “postcuff” reflects the maximal or minimal value after deflation, and “end cuff” reflects the final 15 s of the post-cuff period.

### Reporting summary

Further information on research design is available in the Nature Research Reporting Summary linked to this article.

## Supplementary information

Reporting Summary Checklist

## Data Availability

Data sharing is subject to the conditions of approval by the relevant ethics boards, and therefore cannot be available without appropriate consent.
